# A National Population Study of the Co-Occurrence of Multiple Long-Term Conditions in People With Multimorbidity, Denmark, 2013

**DOI:** 10.5888/pcd13.150404

**Published:** 2016-01-28

**Authors:** Karina Friis, Marie Hauge Pedersen, Finn Breinholt Larsen, Mathias Lasgaard

**Affiliations:** Author Affiliations: Marie Hauge Pedersen, Public Health and Quality Improvement, Central Denmark Region, Aarhus, Denmark; Finn Breinholt Larsen, Public Health and Quality Improvement, Central Denmark Region, Aarhus, Denmark; Mathias Lasgaard, Public Health and Quality Improvement, Central Denmark Region, Aarhus, Denmark, and Department of Psychology, University of Southern Denmark, Odense, Denmark.

## Abstract

The objective of this study was to describe the prevalence of pairwise combinations of 17 long-term conditions. Data were obtained from a national, representative population-based study including 162,283 Danish citizens aged 16 years or older. We calculated the prevalence of each long-term condition given the presence of another long-term condition. Compared with the general population, people with angina pectoris had more than twice the odds of having 12 of the 16 other long-term conditions, and inversely, people with cancer, tinnitus, or cataracts did not have notably higher odds for any of the other long-term conditions.

## Objective

The study objective was to describe the prevalence of pairwise combinations of 17 self-reported long-term conditions. Research on chronic disease clusters at the population level is scarce despite the fact that multiple diseases tend to compound and interact (1). Such research demands large samples, because possible combinations of long-term conditions are numerous, and the occurrence of some diseases is rare. Furthermore, most research on the clustering of diseases includes only the elderly population (2–5). Studies of combinations of long-term conditions are relevant to clinical practice guideline committees who define the standard of care for single conditions and comorbidities.

## Methods

The present study was based on data from the Danish 2013 National Health Survey, *How Are You?* Of 300,450 randomly selected Danish citizens aged 16 years or older invited to participate, 162,283 (54.0%) completed the questionnaire.

Data on long-term conditions were collected by using a revised version of a survey instrument recommended by the World Health Organization for use in national health surveys (6). Respondents were asked if they had any of 18 long-term conditions or if they had had any of the long-term conditions in the past and were still affected by one or more of the following conditions: asthma, allergy, diabetes, hypertension, myocardial infarction, angina pectoris, stroke, chronic obstructive pulmonary disease (COPD), osteoarthritis, rheumatoid arthritis, osteoporosis, cancer, migraine or recurrent headaches, mental disorders for 6 months or less, mental disorders for 6 months or longer, slipped discs or other back injuries, cataract, or tinnitus. To enhance data quality, the 2 questions on mental disorders were combined into one category before data analysis. The present study, therefore, considered 17 conditions.

The unique personal identification number registered in the Danish Civil Registration System was used to link respondents and nonrespondents to the national registers. Weights were constructed by using a model-based calibration approach, and because of these weights, data were considered to be representative of the Danish population.

Missing data did not exceed 1.5% for any of the variables, and missing data were not excluded before any of the analyses. We calculated the prevalence of 272 pairwise combinations of the 17 long-term conditions adjusted for age and sex. Contrast analysis was performed to compare the prevalence of a long-term condition at the population level with its prevalence in a specific dyad; odds ratios were adjusted for age and sex and categorized by magnitude (≤2.0, >2 and ≤3.0, and >3.0). Age was treated as a continuous variable. We performed 272 tests. Applying a Bonferronni correction, we evaluated all statistical tests at the 0.00018 probability level (0.05/272).

## Results

Thirty-eight percent of the population had none of the 17 long-term conditions, whereas 29% had one condition, and 33% had 2 or more conditions. The Figure shows the percentage prevalence of each condition at the population level and the age- and sex-adjusted prevalence of each condition given the presence of another condition. 

**Figure:**
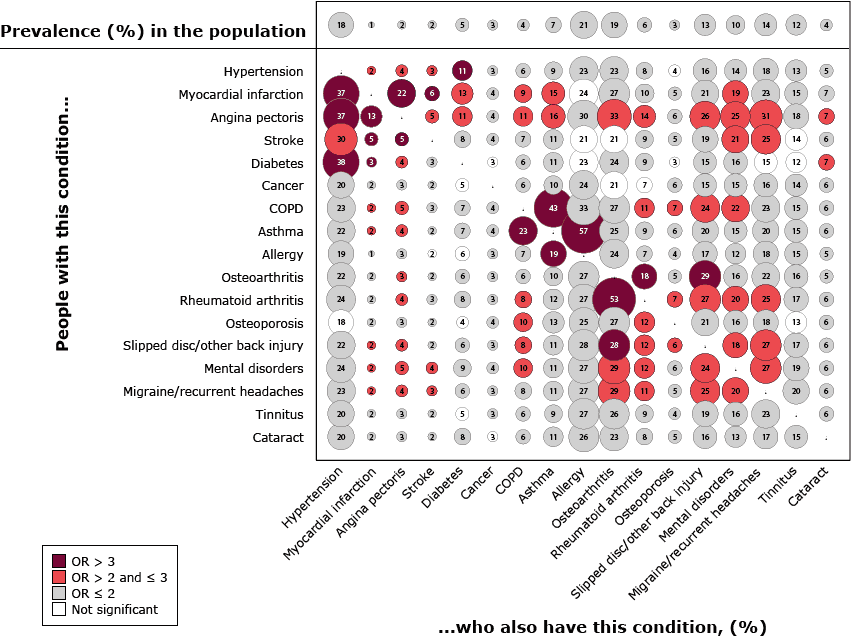
Unadjusted percentage prevalence of 17 long-term conditions and age- and sex-adjusted percentage prevalence in each pairwise combination among the Danish population aged 16 years or older who responded to the Danish 2013 National Health Survey, *How Are You?* Odds ratios compare prevalence of the condition in each pair with overall prevalence of the condition. Numbers inside the bubbles indicate percentage prevalence for each pair. Size of circles indicates prevalence value: the larger the circle, the greater the prevalence. Bubble colors indicate how the age- and sex-adjusted disease-specific prevalence relates to the age- and sex-adjusted population prevalence. Abbreviations: COPD, chronic obstructive pulmonary disorder; OR, odds ratio.

At the population level, the prevalence of each condition ranged widely, from myocardial infarction (1%) to allergies (21%). In 252 of the 272 combinations, the disease-specific prevalence was significantly larger than the prevalence in the general population. Several conditions were twice as prevalent in a combination as they were in the general population. The 3 most common dyads were allergy (57% of asthmatics), osteoarthritis (53% of people with rheumatoid arthritis) and asthma (43% of people with COPD).

 Several conditions were more likely than other conditions to be found in multiple high-prevalence pairwise combinations. For example, compared with people in the general population, people with angina pectoris were more than twice as likely to have 12 of 16 other conditions (Figure). Having a slipped disc or other back injury or a mental disorder was also associated with a higher likelihood of having 8 of 16 other conditions. Inversely, having cancer, tinnitus, or cataracts was not associated with higher odds for having any of the other conditions.

## Discussion

This study showed that the most common combinations of long-term conditions were allergy and asthma, osteoarthritis and rheumatoid arthritis, and asthma and COPD. Managing a cluster of such conditions with synergistic management strategies (eg, concordant conditions like asthma and allergy) is probably less difficult than dealing with a combination of conditions with nonsynergistic management strategies (eg, discordant conditions like angina pectoris and COPD) (7). Particularly noteworthy is the high prevalence of somatic conditions (eg, COPD, angina pectoris) among people with mental disorders. People with mental disorders have a higher mortality rate than people without mental disorders, and cardiovascular disease is a major contributing factor (8,9).

To the best of our knowledge, this is one of the first population-based studies to examine the prevalence of a long-term condition in the presence of another long-term condition across a broad age span. However, several studies have illustrated that many long-term conditions tend to co-occur (1,2,10–12).

The main strength of our study is that it used data from a large, national, representative sample. However, because the data were cross-sectional, no conclusions about temporality or causation can be made. Furthermore, all conditions were self-reported, and no objective verification of the self-reported diagnoses was possible. However, using self-reported data allowed us to obtain information about conditions such as allergies, musculoskeletal diseases, and migraine, which is rarely possible in studies that rely solely on registry data. A third limitation is that the study did not account for complex patterns of disease co-occurrences.

Our results may help clinicians identify synergistic and nonsynergistic multimorbidity scenarios commonly encountered in clinical practice.
